# A Case Study: Comprehensive Approach for Treating Horizontal Neck Wrinkles Using Hyaluronic Acid Injections and Thread-Lifting

**DOI:** 10.1007/s00266-022-03071-7

**Published:** 2022-08-25

**Authors:** Zhi-Feng Liao, Wei Yang, Fu-Chuan Lin, Shi-wei Wang, Wei-Jin Hong, Sheng-Kang Luo

**Affiliations:** 1 Department of Plastic and Reconstructive Surgery, Guangdong Second Provincial General Hospital, 466 Middle Xin Gang Road, Guangzhou, 510317 Guangdong Province People’s Republic of China; 2 Beijing Huaxia Medical Beauty Clinic, Beijing, People’s Republic of China; 3 Department of Medical Affairs, Imeik Technology Development Co., Ltd, Beijing, People’s Republic of China

**Keywords:** Horizontal neck wrinkles, Hyaluronic acid injections, Thread-lifting, Neck rejuvenation

## Abstract

**Background:**

Horizontal neck wrinkles develop during the aging process.

**Aims:**

This study assessed the effectiveness of a comprehensive approach to treating horizontal neck wrinkles using non-cross-linked hyaluronic acid injection and smooth absorbable PPDO (Poly p-dioxanon) thread insertion.

**Methods:**

Ten patients with horizontal neck wrinkles were treated with hyaluronic acid injection and thread-lifting. The clinical outcomes were evaluated six months after treatment.

**Results:**

The median global aesthetic improvement scale scores evaluated by plastic surgeons and the patients were 4.3 ± 0.8 (3–5) and 4.1 ± 0.7 (3–5), respectively, at six months post-treatment. Five (50%) patients strongly agreed, and three subjects (30%) agreed that their horizontal neck wrinkles had improved following treatment. No serious adverse events, including infections, lumps, irregularities, or the Tyndall effect, occurred during treatment.

**Conclusion:**

This study revealed that a comprehensive approach using hyaluronic acid and thread-lifting provided satisfactory and effective clinical outcomes in treating horizontal neck wrinkles.

**Level of evidence IV:**

This journal requires that authors assign a level of evidence to each article. For a full description of these Evidence-Based Medicine ratings, please refer to the Table of Contents or the online Instructions to Authors http://www.springer.com/00266.

## Introduction

A slender, smooth, wrinkle-free neck is a desirable feature associated with youth and beauty [1]. Horizontal neck wrinkles have been acknowledged as a key feature of the aging neck [2]. It is inevitable that the proportion of collagen fibers and fibroblasts located in the skin dermis gradually decreases as people age. A decrease in the volume of the dermal matrix can result in the appearance of neck wrinkles [3]. Also, overactivity and loss of tension of the platysma muscle can produce vertical muscle bands as well as horizontal neck wrinkles. An increasing number of patients are seeking effective treatments to reduce or remove neck wrinkles resulting from the aging process. Current approaches to neck rejuvenation include laser treatment, injections with fillers, calcium hydroxylapatite, thread-lifting, intensity focused ultrasound (IFU), and incobotulinumtoxin A injections [4–8]. To achieve lasting therapeutic effects, a multimodal approach is usually required to treat neck wrinkles [9]. Recently published articles have evaluated the efficacy of combining several of the strategies mentioned above. However, other combination therapies still need to be explored.

Hyaluronic acid (HA) is an endogenous polysaccharide with the highest concentrations in skin and connective tissue. In the skin, HA polymers bind and retain water molecules that helps with hydration and the maintenance of skin turgor. Therefore, in the aging process, the loss of HA is related to increases in skin dehydration and wrinkles [10–12]. Previous studies demonstrated that injecting HA as a filler into aging skin can restore the hydrobalance and stimulate collagen synthesis, resulting in improved skin structure and elasticity. Therefore, HA filler injection should be an effective treatment to reduce or eliminate horizontal neck wrinkles [13]. Previous studies have reported that the subdermal injection of HA is safe and effective for treating horizontal neck wrinkles. However, the thin skin found in the neck region and the relative lack of fatty tissue increases the risk of producing lumps, irregularities, and the Tyndall effect when HA filler is injected in the neck [14, 15]. Suitable fillers with lower viscosity can result in more comfortable injections, more effective penetration, and higher adhesion, producing better results without the occurrence of nodules or the Tyndall effect.

Suture suspension, also known as thread-lifting, was pioneered by Suramanitz and his colleagues in the 1990s and is popular among dermatologists and plastic surgeons [16]. For patients with primarily soft tissue ptosis and less skin redundancy, thread lifting is a preferred option. The use of facial sutures and suspension is not a new surgical method. It is popular because it is minimally invasive with few adverse events; it does not involve skin excision, superficial myoaponeurotic system (SMAS) flaps, or the removal of excess skin [17, 18]. Based on the materials used for suturing and the filling line, the thread materials can be divided into absorbable and non-absorbable materials. Recent studies carried out using non-absorbable sutures have revealed that thread-lifting may be a good choice compared to more invasive procedures. However, non-absorbable sutures remain in the tissue, which can result in complications, including palpable irregularities in the skin and occasional sutures that protrude through the skin. Therefore, it is necessary to use an absorbable PPDO thread in this procedure [19, 20].

In this paper, we described a novel approach to treat horizontal neck wrinkles using hyaluronic acid and thread-lifting. We also discussed the safety and efficacy of this novel approach.

## Materials and Methods

### Patients

Ten patients with a Wrinkle Assessment Scale (WAS) [21] of 2–5 for horizontal neck wrinkles were enrolled in this study, including one male and fifteen females. The average age of the patients was 42.6 ± 11.8 years (range, 22–56 years).

Patients with bleeding tendencies, coagulation disorders, severe diabetes, hypertension, hypertrophic or keloid scars, hypersensitivity to any of the components of injected non-cross-linked hyaluronic acid or other systemic diseases were excluded from this study. Also, patients who had previously undergone neck laser therapy, chemical peeling, botulinum toxin injections, thread implantation, soft tissue material filling, or surgery were excluded.

The study protocol was approved by the Institutional Review Board of the Guangdong Second Provincial General Hospital. All patients provided written informed consent. A single surgeon performed all the procedures.

### Preoperative Preparation

Before thread-lifting, all patients underwent routine physical examinations and photographic evaluation. All markings were completed with the patient in a sitting position. Topical anesthesia (8% lidocaine cream) was applied 30 to 60 minutes before treatment to the areas to be treated.

### Thread-Lifting

**Fig. 1:**
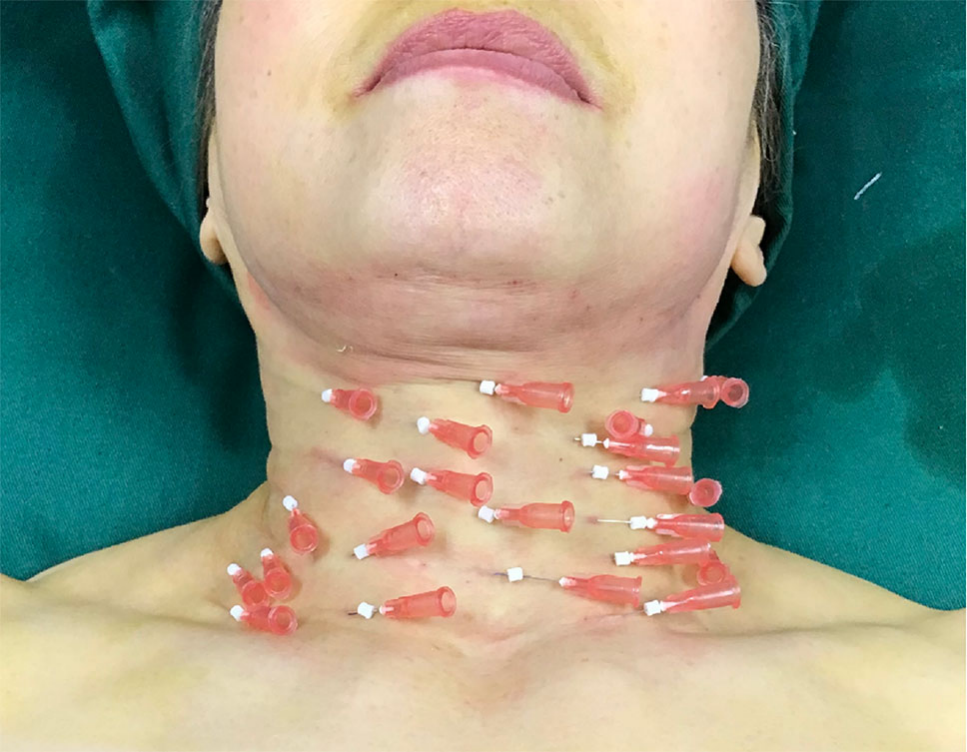
Schematic view of tread lifting of anterior view of the neck

The suture material used in this study was a type of smooth absorbable PPDO (Poly p-dioxanon) thread manufactured by the IMEIK Technology Development Co., Ltd. (Beijing, China). Patients were placed in the supine position to expose the anterior neck and wrinkles. After the asepsis and antisepsis measures were completed, the incision site and insertion areas were injected with local anesthesia (lidocaine, 1%, and adrenaline, 1:200,000), for analgesia and hemostasis, respectively. A 5/0 PPDO thread with a length of 5.0 cm was inserted into the subcutaneous plane using a guide cannula with the bevel up and an insertion angle of 10 to 15 degrees to the skin surface. In the process of inserting the needle, the surgeon’s right hand guided the cannula forward, and the left hand continuously explored the subcutaneous layer with the embedding needle to ensure that the sutures were threaded in the subcutaneous tissue layer uniformly (Figure 1). After completing the first thread-lifting, another was initiated. Sutures were threaded in the subcutaneous tissue plane from one side of the neck wrinkle to the other side, using a serial lifting technique. The number of threads that were placed was determined by the number of neck wrinkles present in each patient.

### Hyaluronic Acid Injection

**Fig. 2:**
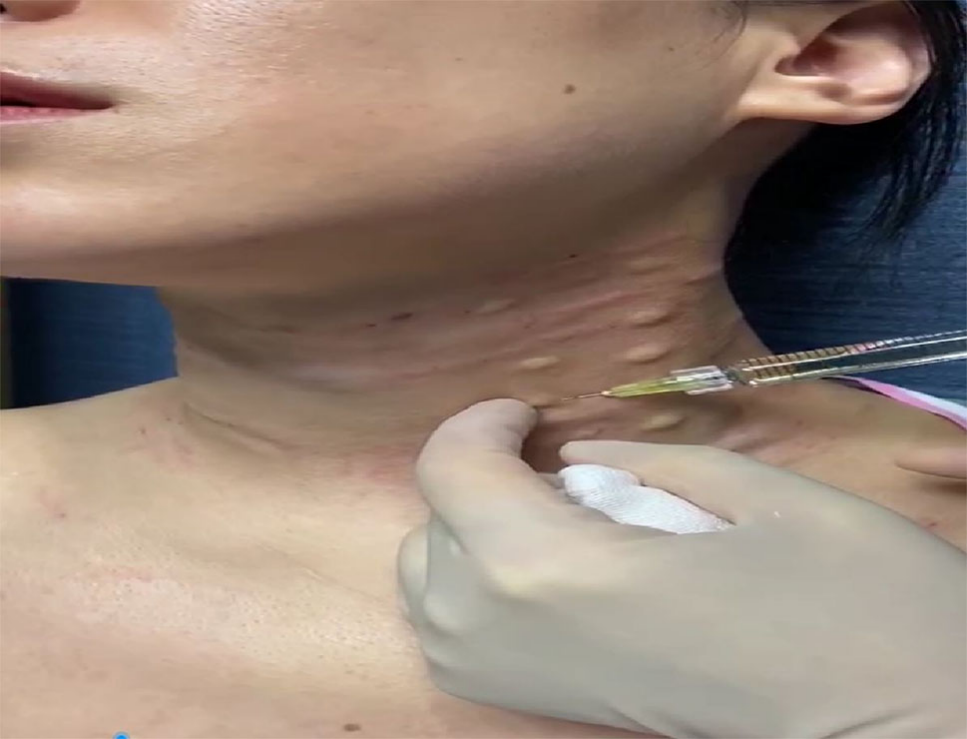
Schematic view of Hyaluronic Acid injections of oblique view of the neck

**Fig. 3:**
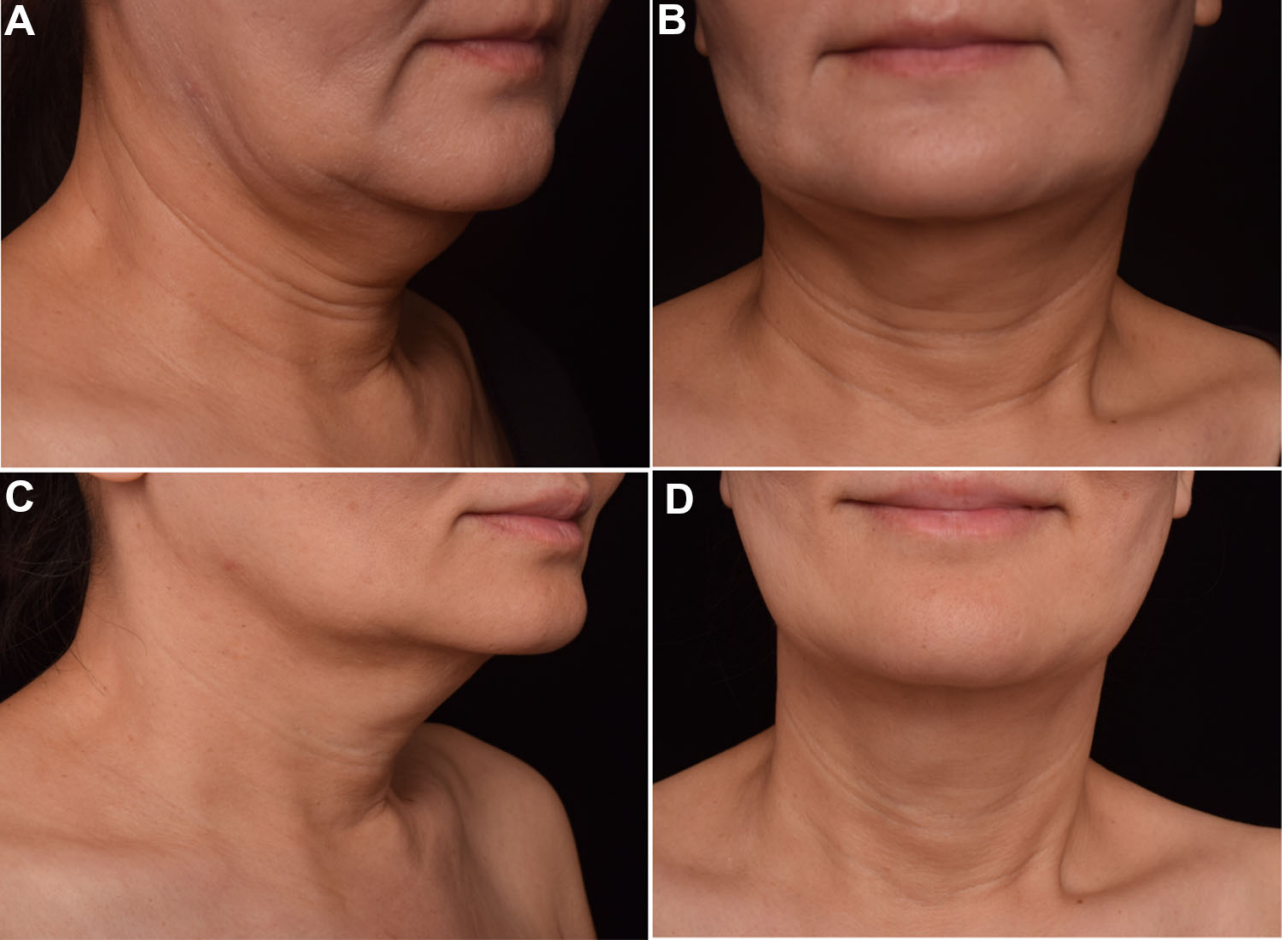
Patient 1 who received a total 5.5ml HA injection and 5/0 PPDO thread lifting. Oblique view (**A**) and anterior view **B** of clinical photographs at baseline and at 6 month after treatment (**C** and **D**)

**Fig. 4:**
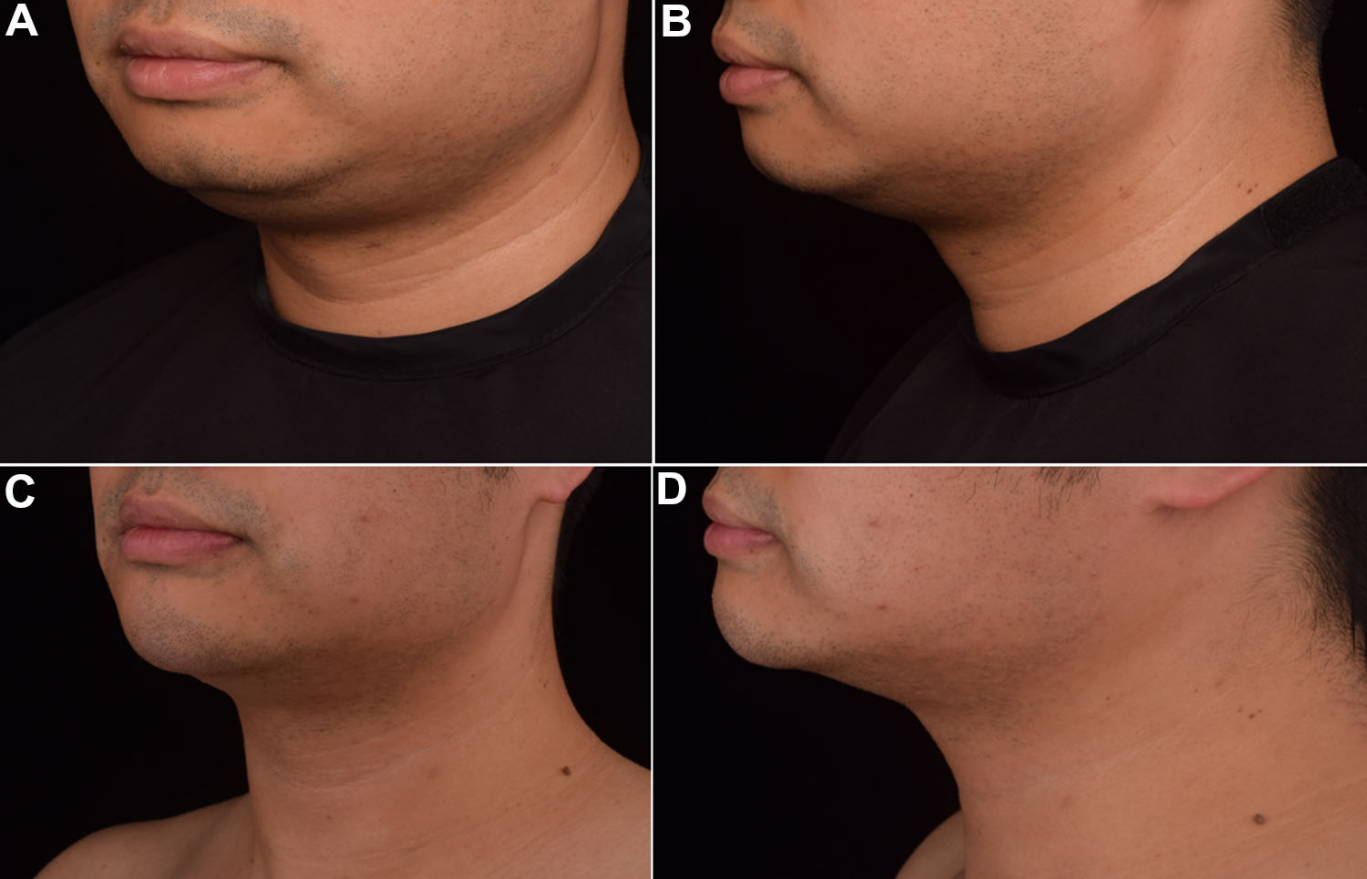
Patient 2 who received a total 5.5ml HA injection and 5/0 PPDO thread lifting. Oblique view (**A**) and anterior view **B** of clinical photographs at baseline and at 6 month after treatment (**C** and **D**)

**Fig. 5:**
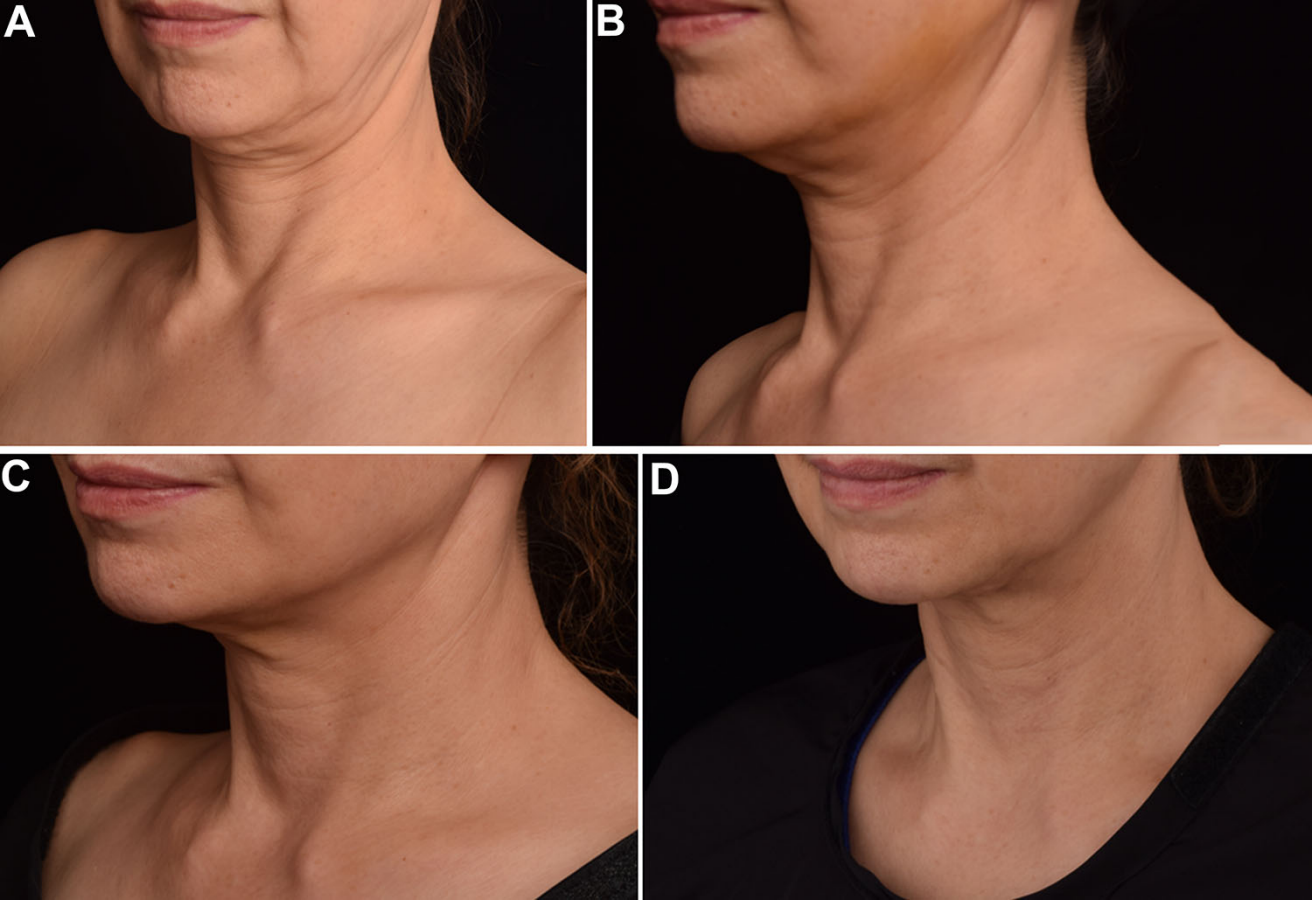
Clinical photographs of patient 5. Clinical photographs at baseline (**A**) and 2 month (**B**), 4 months (**C**), 6 months **D** after multimodal treatment using HA injection at a total volume of 8 mL and 5/0 PPDO thread lifting

The HA injection was performed immediately following the completion of the thread-lifting. The filler (named as Hearty, manufactured by the IMEIK Technology Development Co., Ltd.) used in this study was a sodium hyaluronate composite solution suitable for injection and consisted of non-cross-linked hyaluronic acid, amino acids, vitamins, and other components. Two different solution specifications (1.5 ml and 2.5 ml) were used. The 1.5 ml HA, with a high molecular weight, was used in patients with deep neck wrinkles. A 30-gauge needle was fully inserted into each horizontal neck wrinkle using an insertion angle of 10 to 15 degrees to the skin surface and with the bevel up (Figure 2). Injections were made into the deep dermis along the horizontal neck wrinkles. The needle was withdrawn using the linear threading technique. A single injection delivered 0.05 to 0.1 ml of the filler solution over a length of 8 to 10 mm. For these patients, a superficial serial puncture technique, with punctures spaced 0.5 cm apart, was used along the horizontal neck wrinkles (Figures 3, 4 and 5). A volume of 0.01 to 0.02 mL/puncture was injected into the reticular dermis. The amount of filler solution used varied according to the depth, length, and number of wrinkles for each patient. After every three to five injections, gentle massage and pressure were applied to the injection sites. Patients received up to four treatment sessions using the injected HA filler solution at one-month intervals.

The average duration of the procedures was 60 to 90 minutes. After completing the combined treatment, the treated area was cooled with ice packs to reduce swelling and edema. Patients were advised against carrying out vigorous neck exercises for seven days. Melilotus extract tablets (100mg) were given three times a day for four days. No antibiotics were administered prophylactically to the ten patients.

### Objective and Subjective Clinical Assessment

Photographs were taken in oblique view and anterior view at the baseline examination (pre-injection) and six months post-treatment. Each subject was photographed under standard conditions that included the same photographer, consistent camera settings, standing posture, and uniform lighting.

### Global Aesthetic Improvement Scale (GAIS)

At six months post-treatment, two plastic surgeons who had no conflicts and did not participate in the study procedures objectively evaluated the overall improvement of the patients’ horizontal neck wrinkles. Specifically, they each analyzed the pictures of the patients before and after treatment and carried out their assessments based on the Global Aesthetic Improvement Scale (5‐very much improved, 4‐much improved, 3‐improved, 2‐no change, 1‐worse). In addition, the patients were asked to subjectively evaluate the overall improvement of their horizontal neck wrinkles using the GAIS at six months post-treatment.

### Likert Response Scale

Patients’ satisfaction at six months post-treatment was evaluated using the 13-item patient satisfaction questionnaire that was graded using a five-point Likert response scale (1 = strongly agree, 2 = agree, 3 = neither agree nor disagree, 4 = disagree, and 5 = strongly disagree). Throughout the study, participants were asked to report any adverse symptoms that they experienced and record the duration of any adverse events.

## Results

The clinical outcomes were objectively assessed through the use of the GAIS, which included assessing the length, width, and severity of the horizontal neck rhythids, in addition to the skin texture and the severity of the vertical platysmal bands. The median GAIS determined by the plastic surgeons were 4.3 ± 0.8 (with a range of 4 to 5) at six months post-treatment, while the median GAIS evaluated by the patients was 4.1 ± 0.7 (with a range of 4–5) (Table 1).

**Table 1:** Overview of demographics, interventions and effective assessments data.

Patient NO.	Age (Years)	Sex	GAIS at 6 month		Patient satisfaction
			Surgeon	Patient	
1	55	F	5	5	1
2	33	M	4	4	2
3	22	F	3	3	3
4	32	F	4	5	1
5	56	F	5	4	2
6	50	F	5	5	1
7	49	F	4	4	2
8	39	F	5	4	1
9	36	F	3	3	3
10	54	F	5	4	1

Using the Likert response scale, five (50%) patients strongly agreed that their horizontal neck wrinkles were diminished, and three (30%) patients agreed that their horizontal neck wrinkles were diminished. Two (20%) patients neither agree nor disagree that their horizontal neck wrinkles were diminished.

No serious adverse events, including infections, lumps, irregularities, or the Tyndall effect, occurred during the course of treatment. Mild adverse events, including erythema, ecchymosis, and localized swelling, were reported by some patients, but all events were transient and resolved spontaneously within two weeks.

## Discussion

In this study, the authors evaluated the clinical efficacy and safety of a comprehensive approach to treat neck wrinkles using HA and thread-lifting. The present data revealed that at six months post-treatment, the clinical improvement scores determined by the GAIS from the surgeons and subjects were 4.3 and 4.1, respectively. Also, all ten patients were satisfied with the improvement that occurred in their horizontal neck wrinkles. Three patients only reported mild bruising, pain and redness following the filler injection and thread lifting, which are widely considered the common AEs of injection and thread lifting, and the AEs lasted only for a short duration (7 days) and were resolved with a gentle ice compress. Within the six months of follow-up, no serious adverse events, including infections, lumps, irregularities, or the Tyndall effect, occurred.

The horizontal neck fold was the first sign of aging, the aging process alters the

mechanical properties of the skin and subcutaneous fat cause by photoaging, primarily by decreasing the collagen and elastic fibers in the dermis and volume loss. As these fibers become thinner and separated, the skin generally loses elasticity, and therefore, forms wrinkles [22]. So, in this study, we applied the smooth absorbable PPDO to fill the subcutaneous layer.

The use of thread-lifting for face or neck suspension is not new and has been used to achieve nonsurgical rejuvenation, with minimal invasion and fewer perioperative complications. Thus, thread-lifting is of interest to patients, is a preferred method for surgical lifting among surgeons, and is widely used for face or neck rejuvenation [23]. The thread materials can be divided into absorbable and non-absorbable categories. Non-absorbable threads are permanently retained in the tissue, which might lead to complications, including skin dimpling, foreign body reactions, infection, and extrusion. For these reasons, the use of non-absorbable threads is gradually decreasing, and thread-lifting using absorbable threads has become the preferred method [20].

The PPDO threads used in this study were a type of absorbable smooth thread similar to polydioxanon (PDO) thread. Compared with PDO sutures, PPDO suture material retains excellent biocompatibility and degradability, and it also has higher tensile strength and greater flexibility. However, due to the smooth thread surface and the absence of any barbs or serrations, the PPDO thread has a limited ability to lift flabby tissue. Thus, these smooth sutures are more suited for filling depressions and improving wrinkles because of their physical filling effect and stimulation of collagen and elastic fiber formation. Also, thread-lifting using smooth suture facilitates a more rapid and straightforward procedure and minimizes tissue injury. Our results demonstrated that the use of PPDO thread to improve neck wrinkles was a safe and effective treatment method.

When the threads are inserted into the subcutaneous tissue layer, it produces an immediate volume enhancement due to the thread volume and the induced swelling. Subsequently, the thread continues to exert effects on the tissue. Many histopathological studies have indicated, the use of absorbable lifting threads might trigger TGF-b signaling, which induces increases in collagen components and fibrosis formation. Therefore, the suture placement enhances the volume of the subcutaneous tissue over time and helps to permanently alter the surrounding structures biologically, resulting in favorable rejuvenation effects, skin lifting, and generating a longer-lasting smooth, contoured appearance [16, 23, 24].

The ability to retain water and the dermal matrix volume in the skin is decreased as people age due to reductions in the concentration of HA in the skin, which increases the tendency for wrinkle formation. Due to its high biocompatibility, reversibility, and high hydrophilicity, HA fillers are thought to be an effective method to treat neck wrinkles. However, thin skin and the presence of relatively little fatty tissue in the neck increase the risk of producing lumps, irregularities, and the Tyndall effect when HA filler is injected in the neck region. Therefore, choosing fillers with suitable rheology and lower viscosity is crucial.

The filler used for injection in this study was a sodium hyaluronate composite solution composed of non-cross-linked HA, amino acids, vitamins, and other components. It has been demonstrated that injection of a non-cross-linked HA into the skin can provide a volume-filling effect and induce collagen synthesis, which results in a noticeable decrease in neck wrinkles. It also restores skin hydrobalance and effectively improves skin texture, brightness, and elasticity. With greater fluidity and lower viscosity, the use of this type of HA filler results in more comfortable injections and can more uniformly integrated into the dermis to create a soft, diffuse correction, which minimizes the risk of lump formation or irregularities in the skin. Thus, this type of HA filler selection is highly suitable to fill horizontal neck wrinkles.

Nowadays, widely applied cosmetic HA fillers are mainly cross-linked, which is less appropriate for neck wrinkles because of its thin dermal layer and less subcutaneous fat tissue. Nodules are reported cross-linked HA injection for correcting neck wrinkles. Wang [25] had our proved the short-term efficacy and safety of non-cross-linked HA for mild-to-severe horizontal neck wrinkle. Besides, the non-cross-linked HA could immediately smoothen the neck folds and L-carnosine could possibly prolong the duration of efficacy possibly by reducing the UV-related damage and promoting the collagen regeneration.

Considering the efficacy and safety together, we believe this combination of non-cross-linked HA injection and smooth absorbable PPDO insertion can prevent treatment-related complications and actively prolongs the efficacy of the filler by improving local tissue protecting and repairing activity. However, being restricted by the lack of control group, it was difficult to design this study as a parallel comparative one, to better investigate HA injection and PPDO thread lifting function, respectively. Besides, the small patient sample size and short follow-up duration are also limitations of the study. And further studies should be conducted to investigate a long-term effectiveness of combination of non-cross-linked HA injection and smooth absorbable PPDO insertion.

In conclusion, our results indicated that the combined treatment with HA filler injections and thread-lifting was a safe and effective treatment to reduce or eliminate horizontal neck wrinkles. However, additional studies with control groups and larger sample sizes are needed to confirm our results.
